# Efficacy of an exercise intervention for employees with work-related fatigue: study protocol of a two-arm randomized controlled trial

**DOI:** 10.1186/s12889-015-2434-6

**Published:** 2015-11-12

**Authors:** Juriena D. de Vries, Madelon L. M. van Hooff, Sabine A. E. Geurts, Michiel A. J. Kompier

**Affiliations:** Behavioural Science Institute, Radboud University, P.O. Box 9104, 6500 HE Nijmegen, The Netherlands

**Keywords:** Work-related fatigue, Burnout, Running, Exercise intervention, Employee well-being, Randomized controlled trial

## Abstract

**Background:**

The aim of the current study is to evaluate the efficacy of an exercise intervention to reduce work-related fatigue. Exercise is a potentially effective intervention strategy to reduce work-related fatigue, since it may enhance employees’ ability to cope with work stress and it helps to detach from work. However, based on available research, no clear causal inferences regarding its efficacy can be made. This RCT therefore investigates whether exercise is effective in reducing work-related fatigue, and in improving other indicators of employees’ mental and physical well-being and performance.

**Methods/design:**

A two-arm parallel trial will be conducted. Participants (*N* = 108) who experience high levels of work-related fatigue will be randomized at a 1:1 ratio to a 6-week exercise intervention or wait list (control). The exercise intervention consists of three one-hour low-intensity outdoor running sessions a week. Each week, two sessions take place in a group under supervision of a trainer, and one session is completed individually. The running sessions will be carried out during leisure time. The primary outcome is work-related fatigue. Secondary outcomes include work ability, self-efficacy, sleep quality, cognitive functioning, and aerobic fitness. These data will be collected at pre-intervention, post-intervention, and at 6 weeks and 12 weeks after the intervention. In addition, weekly measures of employees’ well-being, and exercise activities (i.e. type, frequency, and duration) and experiences (i.e. pleasure, effort, and detachment) will be collected during the intervention period.

**Discussion:**

This study will compare an exercise intervention to a wait list. This enables us to examine the effect of exercise on work-related fatigue compared to the natural course of these symptoms. As such, this study contributes to a better understanding of the causal link between exercise and work-related fatigue. If the intervention is proven effective, the results could provide a basis for future ‘effectiveness’ trials in which the (implementation of the) intervention can be investigated among a broader defined population in a less standardized way, eventually leading to better evidence-based policies and practices to employees, employers, health practitioners, and policy makers concerning the effect of exercise on work-related fatigue.

**Trial registration:**

NTR5034. Registered 10 March 2015.

## Background

Work-related fatigue is a global concern (estimated at 22 % among employees in Europe [1]) and is thought to at least partly result from prolonged stress at the work place [2]. Employees who experience work-related fatigue often have poorer work performance [3], are more frequently absent from work [4], and are at higher risk for ill health, such as cardiovascular diseases [5]. If work-related fatigue becomes more severe, it can result in (long-term) clinical burnout [2]. Given the prevalence of work-related fatigue, and the negative impact on employees’ work and health, it is valuable to examine potential intervention strategies to reduce these symptoms.

Regular exercise may be an eligible intervention strategy to reduce work-related fatigue. Assets of exercise include its accessibility, low costs [6], and positive side effects, such as reduced risk for cardiovascular diseases [7].

### Exercise and work-related fatigue

The potential beneficial effects of exercise on work-related fatigue can be understood from a combination of various (interconnected) physiological and psychological mechanisms. For instance, it has been proposed that exercise enhances employees’ ability to (physically and psychologically) cope with work stress [8, 9]. Others found that exercise helps to detach from work [10], and enhances positive affect [11] facilitating employees in replenishing their energy levels. In addition, research indicates that exercise impacts certain neural circuits and the release and uptake of chemicals in the brain, which are associated with better mental health [12, 13].

Despite these proposed theoretical notions, based on available empirical evidence no clear causal inferences can be made about the effect of exercise on work-related fatigue. This is because knowledge about this relation is largely based on correlational studies [14–18], and the relatively few available intervention studies suffered from one or more methodological shortcomings [19–22]. In well-designed intervention studies, exercise was conducted among students [23] (which leaves the question open whether results can be generalized to employees), or exercise was combined with other intervention ingredients [24] (which makes it impossible to know to what extent the beneficial effects were due to exercise). Furthermore, research suggests that it may be more difficult for employees with high levels of work-related fatigue to engage in regular exercise [16–18]. Taken together, there is a need for methodologically sound intervention studies to better understand the causal link between exercise and work-related fatigue.

Against this background, the aim of the current study is to test the effect of exercise on work-related fatigue with a design that allows for strong causal inferences. To this end, employees with high levels of work-related fatigue who currently do not engage in regular exercise will be randomly assigned to either a six-week exercise intervention or a wait list (control group). This enables us to test the effect of exercise on work-related fatigue compared to the ’natural course’ of these symptoms. In the intervention, we will use running as a form of exercise, because it is easy to implement: only running shoes are needed and it is high in flexibility in terms of time and place. Based on the proposed psychological and physiological working mechanisms and available empirical research, we hypothesize:

- *Hypothesis 1*: the exercise intervention is effective in reducing work-related fatigue

Additionally, we will measure the impact of exercise on five secondary outcomes, which are relevant for employees with high levels of work-related fatigue, and which may be expected to be positively affected by means of regular exercise.

### Exercise and self-efficacy

First of all, we will study the effect of exercise on work-related and general self-efficacy. It has been found that employees with high levels of work-related fatigue often experience reduced self-efficacy in their functioning at work [3]. On the other hand, it has been proposed that exercise is accompanied by a sense of mastery, which can increase self-efficacy with respect to exercise [25], and other life domains [26]. Since this current research is carried out in a work context, we will investigate whether exercise benefits work-related self-efficacy. We hypothesize:

- *Hypothesis 2a:* the exercise intervention is effective in improving work-related self-efficacy among employees with high levels of work-related fatigue

As the sense of mastery gained by exercise may also be translated in a general confidence in succeeding at tasks and in situations (i.e. general self-efficacy), we expect:

- *Hypothesis 2b*: the exercise intervention is effective in improving general self-efficacy among employees with high levels of work-related fatigue

### Exercise and sleep

Second, we will study the impact of exercise on employees’ sleep quality, and sleep duration. Sleep quality refers to ‘how well someone sleeps’, and is, for instance, characterized by the number of awakenings during the night and the degree of tiredness upon waking [27]. It has been found that work-related fatigue is negatively related to sleep quality [28]. Concurrently, previous studies have reported that regular exercise improves sleep quality [29]. In accordance with these empirical findings, we expect:

- *Hypothesis 2c:* the exercise intervention is effective in improving sleep quality among employees with high levels of work-related fatigue

Research suggests that exercise may also impact employees’ sleep duration [29]. Various pathways have been proposed to explain this relationship, such as cytokine concentration changes (for a review; see [30]). In agreement with these proposed mechanisms and empirical evidence, we expect:

- *Hypothesis 2d*: the exercise intervention is effective in improving sleep duration among employees with high levels of work-related fatigue

### Exercise and work ability

Third, we will investigate whether exercise benefits work ability. Work ability concerns the physical, psychological, and social capability of an employee to effectively cope with work demands [31]. It has been found that a high level of work-related fatigue is a risk factor for decreased work ability [32]. As exercise contributes to psychological [11], social [33], and physical health [34], it can be expected that exercise enhances employees’ capacity to cope with work demands. Indeed, the scarce available empirical research shows that regular exercise is positively related to work ability [35]. Therefore, we expect:

- *Hypothesis 2e:* the exercise intervention is effective in improving work ability among employees with high levels of work-related fatigue

### Exercise and cognitive functioning

Fourth, the effect of exercise on cognitive functioning will be studied. Research shows that employees with high levels of work-related fatigue often display cognitive deficits, such as impaired executive functioning and cognitive problems in daily life [36, 37]. Executive functioning refers to a set of cognitive brain processes involving mental control and self-regulation [38]. Evidence from a broad range of studies shows that exercise is linked to beneficial changes in cognitive functioning, in particular executive functions [39, 40]. Therefore, we hypothesize:

- *Hypothesis 2f:* the exercise intervention is effective in improving cognitive functioning among employees with high levels of work-related fatigue

### Exercise and aerobic fitness

Fifth, we will examine the effect of exercise on aerobic fitness. Aerobic fitness may be defined as the ability to deliver oxygen to the muscles and to utilize it to generate energy during exercise [41]. Employees with high levels of work-related fatigue are found to have relatively low fitness levels [42]. On contrary, the evidence regarding the positive effects of exercise on fitness is overwhelming [43], but effects also depend on the type, frequency, intensity, and duration of exercise [44]. Because participants in our study will change from not exercising to regular (i.e. 3 times a week) exercising, we hypothesize:

- *Hypothesis 2 g:* the exercise intervention is effective in improving aerobic fitness among employees with high levels of work-related fatigue

For all primary and secondary outcomes (except cognitive functioning, due to possible learning effects), we will include follow-up measures to examine whether the proposed beneficial effects of exercise last over time.

### Weekly trajectories of employee well-being

Next to measuring the effect of exercise on these primary and secondary outcomes after the intervention period, it is also valuable to examine employees’ well-being trajectories during the intervention period. This prevents the intervention period of being a ‘black box’, in which it remains unknown which changes take place at what time. Therefore, in the current study, we will pay close attention to employee well-being during the intervention period. We will employ weekly self-administered single-item measures of fatigue, self-efficacy and sleep, which correspond to our primary and secondary outcomes. Additionally, we will investigate other single-item indicators of employee well-being, such as mood, to capture a broader range of the effects of the intervention. By doing so, we aim to provide a more fine-grained insight in how these different indicators of employee well-being develop during the intervention period, and whether weekly trajectories of these indicators differ between conditions. Although it is not possible to predict at what time these indicators change as a result from the intervention, we generally expect that:

- *Hypothesis 3*: during the course of the intervention period, participants in the exercise condition show a larger improvement in weekly well-being compared to participants in the control condition.

### Compliance, and subjective exercise experiences as moderators of employee well-being

Finally, we will investigate possible moderators in the relation between exercise and employee well-being: compliance to the intervention and subjective exercise experiences. This could give us information for whom or under what conditions the intervention works best. Concerning compliance, we will measure participants’ compliance with the running sessions of the exercise intervention. Participants who attend more of the running sessions of the intervention will likely also benefit more from the beneficial changes brought about by exercise. Therefore, we expect:

- *Hypothesis 4a*: during the course of the intervention period participants with higher attendance rates show larger improvement in weekly well-being compared to participants with lower attendance rates

It has been argued that not only the type of activity (i.e. exercise) matters for beneficial effects on employee well-being, but that also the subjective experience of the activity is important [45, 46]. Therefore, we will pay attention to three ratings of employees’ experiences (i.e. ‘pleasure’, ‘detachment’, and ‘effort’) during the running sessions, and examine how these experiences may or may not moderate exercise intervention efficacy. As research has shown that effects of activities, such as exercise, on well-being are stronger when these activities are enjoyed [46, 47], we expect in this current study:

- *Hypothesis 4b:* participants who rate their weekly running sessions as pleasant show a larger improvement in weekly well-being than participants who rate these sessions as less pleasant

Furthermore, as it has been found that employees who during activities experience psychological detachment from work (i.e. mentally distancing oneself from work), show better well-being outcomes [45], we hypothesize:

- *Hypothesis 4c:* participants who psychologically detach from work during their weekly running sessions show a larger improvement in weekly well-being than participants who cannot psychologically detach from work during these sessions

For the experience of effort, it is difficult to formulate a hypothesis. Although exercise is an effortful activity in nature, research suggest that a too high intensity of exercise could lead to unbeneficial outcomes in well-being [48]. We will therefore investigate the experience of effort in relation to the weekly well-being outcomes in a more exploratory way.

## Methods/design

### Study design

**Fig. 1:**
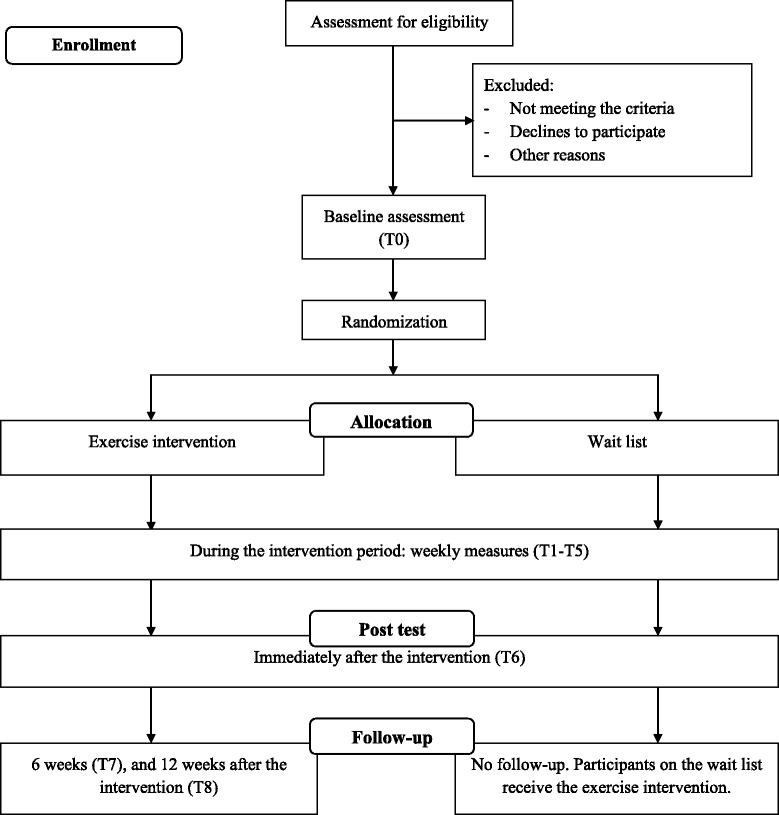
Flow Diagram Study Protocol

The study design will be a two-arm parallel randomized controlled trial. The effect of an exercise intervention on work-related fatigue will be compared to wait list. See Fig. 1 for an overview of the study design.

### Ethical issues

The research plan has been approved by the Ethics Committee Faculty of Social Sciences of the Radboud University (registration number: ECSW2015-1901-278). Informed consent for participation in the study will be obtained from all participants.

### Participants and recruitment

Participants will be employees reporting high levels of work-related fatigue. Every employee – regardless job position – can sign up for participation. Participants will be recruited through advertisements with study information in personnel magazines, on facebook pages, and on intranet of large organizations in the region(s) in which the intervention will be carried out. Furthermore, advertisements and news items will be placed in (local) newspapers and on social media.

Inclusion criteria will be based on existing cut-off scores on two validated measures of work-related fatigue: a) ≥2.2 on the emotional exhaustion scale of the Dutch version of the Maslach Burnout Inventory [49]; b) ≥22 on the Fatigue Assessment Scale [50]. Participants can fill out these two scales on the study website (www.runtervention.nl). When participants score high on both scales, the researcher (JdV) will ask these participants for further information about exclusion criteria by email. Exclusion criteria will be: a) ≥ one hour exercising a week; b) fatigue attributable to a medical condition; c) currently or in the past six months receiving psychological and/or pharmacological treatment; d) drug dependence; e) contraindications to exercise. Contraindications to exercise will be measured by the Physical Activity Readiness Questionnaire (PAR-Q; [51]). Eligible participants will be invited for an appointment with the first author (JdV) or research assistants. During this appointment, the baseline measures (T0; except for the two measures of work-related fatigue that are already filled out) will be completed, the cognitive performance tests will be done, and the randomization procedure will be carried out. A separate appointment will be planned for the aerobic fitness test.

### Randomization and blinding

Participants will be randomly assigned to either an exercise condition or a wait list condition at a 1:1 ratio. Because the intention is to conduct the exercise intervention in groups of 10 participants, randomization will be based on a block size of 20. When the number of 20 eligible participants in a block is reached, participants in the exercise intervention condition start the intervention and the participants in the wait list condition remain on the wait list. The randomization procedure will be carried out after the participant has completed the baseline measurement (T0). The randomization procedure will be executed by the first author (JdV) or research assistant, using sealed opaque envelopes. The participant opens the envelope and tells the researcher to which condition (s)he has been assigned.

### Conditions

#### Exercise intervention condition

The exercise intervention will take six consecutive weeks and comprises of low intensity running, meaning that participants should be able to talk while running [52]. This intensity is chosen, because research has indicated that a low intensity is effective in reducing fatigue [48] and to reduce the risk of injuries [53]. Furthermore, the prospect of high intensity exercise may hamper participants’ motivation to engage in or maintain exercise [54], especially when participants are already fatigued. In total, participants will run three times a week (18 running sessions in total): twice a week in a group of ten people guided by a running trainer, and once a week independently. Each running session lasts one hour, and consists of 15 min warming up, 30 min running alternated with walking, and 15 min cooling down. During the intervention, a fixed running schedule will be used in which the periods of running gradually increase, and the periods of walking gradually decrease. The goal is to achieve a 20 min period of continuous running in the last session. Each week, participants receive the running schedule of the upcoming week by email, as to know the content of the running sessions of that week beforehand. If participants miss a guided running session, they can catch up by doing an additional independent session in the same week that the guided session was originally planned. Participants will be advised to keep at least one rest day (i.e. no running session) in between the running sessions to reduce the risk of injuries [55].

Trainers of the exercise intervention will be experienced running trainers, additionally trained in the principles of ‘running therapy’, the most commonly applied form of exercise therapy for mental health problems in the Netherlands [56, 57]. Trainers will be instructed to watch participants’ level of exertion during the running sessions and to urge participants to lower their speed if they are short of breath and not able to talk. Further, they will be instructed to ensure that the focus during the running sessions is not on ‘performance’ but rather on ‘pleasure’.

### Wait list condition

During the six-week exercise intervention condition, the participants in the wait list condition receive no intervention. After these six weeks, participants will receive the exercise intervention as well.

### Measures

An overview of the measurement points of the primary outcome, secondary outcomes and weekly measures is given in Table 1. All measures, except for the cognitive performance tests and the aerobic fitness test, are self-administered (online questionnaires, sent by email). Completion of the self-administered questionnaires at T0, T6, T7, and T8 will take about 10 min. Participants in the wait list condition will complete the same measures as the participants in the exercise condition (T0-T6), except for the measures of subjective exercise experiences, compliance to the running sessions, and the follow-up measures at 6 and 12 weeks (T7 and T8).

**Table 1:** Overview of the measurement points of the primary outcomes, secondary outcomes and weekly measures

	Construct	Pre intervention (T0)	During intervention (every week) (T1-T5)	Post interventionª (T6)	Follow-up after 6 weeks^b^ (T7)	Follow-up after 12 weeks^b^ T8)
Primary outcome	Work-related fatigue:					
	- Emotional exhaustion	√		√	√	√
	- Need for Recovery	√		√	√	√
	- Overall Fatigue	√		√	√	√
Secondary outcomes	Sleep:					
	- Sleep quality	√		√	√	√
	- Sleep quantity	√		√	√	√
	Self-efficacy:					
	- General self-efficacy	√		√	√	√
	- Work self-efficacy	√		√	√	√
	Work ability	√		√	√	√
	Cognitive functioning:					
	- Updating	√		√		
	- Inhibition	√		√		
	- Switching	√		√		
	- Subjective costs of the tests	√		√		
	- Cognitive Failures	√		√		
	Aerobic fitness:					
	- VO_2max_	√		√		
	- Subjective costs of the test	√		√		
Weekly measures	Employee well-being:					
	- Health	√	√√√√√	√		
	- Mood	√	√√√√√	√		
	- Fatigue	√	√√√√√	√		
	- Tension	√	√√√√√	√		
	- Stress	√	√√√√√	√		
	- Energy	√	√√√√√	√		
	- Satisfaction	√	√√√√√	√		
	- Irritation	√	√√√√√	√		
	- Sleep	√	√√√√√	√		
	- Self-efficacy (sport, work, private)	√	√√√√√	√		
	Exercise activities:					
	-Compliance to running sessions^a^		√√√√√	√		
	- (Other) exercise activities	√	√√√√√	√	√	√
	Subjective exercise experiences:					
	- Pleasure^a^		√√√√√	√		
	- Effort^a^		√√√√√	√		
	- Detachment^a^		√√√√√	√		

^a^Compliance to running sessions and subjective exercise experiences will be only collected among participants in the exercise condition, because participants in the control condition receive no running sessions during the intervention period

^b^Follow-up measures will only be collected among participants in the exercise condition, because at the time of the follow-up measures the participants in the control condition receive the exercise intervention

### Primary outcome measures

#### Work-related fatigue

We will use three self-reported indicators to measure work-related fatigue. First, *emotional exhaustion* will be assessed with the subscale ‘Emotional exhaustion’ of the Dutch adaptation of the Maslach Burnout Inventory [58], the Utrecht Burnout Scale (UBOS [59]). The scale consists of 5 items, for example “I feel burned out from my work” (0 = *never*, 6 = *every day*). A mean score equal to or higher than 2.2 on this scale is defined as a ‘high level’ of work-related fatigue [59]. Previous research shows that psychometric properties of this scale are good [60]. Secondly, *overall fatigue* will be measured with the 10-item Fatigue Assessment Scale (FAS [50]). An example question is “I get tired very quickly” (1 = *never*, 5 = *always*). A sum score higher than 21 is indicative of a high level of fatigue [61]. The scale has found to be valid and reliable [50]. Third, *need for recovery* will be assessed by a short version of the ‘Need for Recovery Scale’ [62]. The scale consists of 6 items. An example item is: “Because of my job, at the end of the working day I feel rather exhausted” (1 = *(almost) never*, 4 = *(almost) always*). For this scale, no norm scores are available. Therefore, inclusion was based on the measures UBOS and FAS. Previous research shows that validity is good [62].

### Secondary outcomes measures

#### Self-efficacy

We will use two indicators to measure self-efficacy. First, g*eneral self-efficacy* will be assessed using a Dutch translation of the 12-item General Self-Efficacy Scale [63]. An example item is: “If I made a decision to do something, I will do it” (1 = *strongly disagree*, 5 = *strongly agree*). Psychometric properties of this scale are good [62]. Secondly, w*ork-related self-efficacy* will be measured by means of the subscale ‘Competence’ of the Utrecht Burnout Scale [59]. An example item is: “If I make plans, I am convinced I will succeed in executing them” (0 = *never*, 6 = *every day*). Validity and reliability of the scale are good [60].

#### Sleep

To measure employees’ *sleep quality*, the 6-item sleep quality scale of the Dutch Questionnaire on the Experience and Evaluation of work will be used [64]. The scale measures the three main components of insomnia (i.e. difficulties in: initiating sleep, maintaining sleep, and restorative sleep) and overall sleep. An example item is: ‘I often wake up several times during the night’ (0 = *no,* 1 = *yes*). Furthermore, participants report their average sleep duration (hours, minutes) as an indicator of *sleep quantity*. Reliability and validity of the scale are found to be good [60].

*Work ability* will be measured by means of a single item [65], namely “Can you indicate how you rate your current work ability when you compare it with your life-time best?” (0 = *completely unable to work*, 10 = *work ability at its best*). This item has been shown to be a good alternative to more comprehensive measures of work ability [65].

#### Cognitive functioning

Employees’ cognitive functioning will be measured by means of four indicators: one self-report measure and three cognitive performance tests.

*Self-reported cognitive problems* will be measured with a Dutch translation of the Cognitive Failure Questionnaire (CFQ [66]). This questionnaire measures the frequency of every day cognitive failures and consists of 25 items. An example question is: “Do you read something and find you have not been thinking about it and must read it again?” (1 = *never*, 5 = *very often*). Previous research shows that the CFQ has excellent psychometric properties [67].

Three types of executive functions (i.e. ‘updating’, ‘switching’, and ‘inhibition’), which are considered as basic executive functions and can be clearly and precisely described [68], will be measured with three validated tests. These tests will only be conducted pre (T0) and post (T6) intervention and not at follow up, because of possible learning effects. The tests will be provided in a counterbalanced order to the participants. However, for each participant the order of tests is similar pre and post intervention. Individual appointments in the lab of the university will be planned. Completion of the three cognitive tests, including filling out the subjective costs, takes about 30 min.

*Updating* will be measured with the 2-Back Task [69]*.* Updating refers to the capability of actively manipulating relevant information in working memory [68]. During the task, 284 stimuli (the letters: b, d, g, p, t, and v) are displayed one by one in the centre of the screen. Each letter will be displayed for 450 ms, and the time between the letters will be 750 ms. The letters are presented in a quasi-randomly order in both capital and small letters. When the displayed letter is similar to the letter that is shown two screens before, participants have to push a button on a button-box (target rate: 32.5 %). For a correct response, no distinction is made between capital and small letters. The number of correct responses will be taken as an indicator of updating.

*Switching* refers to the ability to shift between tasks [68], and will be assessed by the Matching Task [70, 71]. In this task, four different geometric figures (a circle, a hexagon, a square, and a triangle), presented in the colors blue, green, red, or yellow are used as stimuli. The task consists of 31 task runs, each consisting of on average six trials (range: 4–8 trials). During a trial, a colored reference figure is displayed in the upper half of the screen, and four colored match figures are shown in the lower half of the screen. Participants will be instructed to match the reference figure to one of the match figures according to shape or color. Whether participants have to match according to shape or color, will be randomly chosen and indicated by a cue that is displayed for 1000 ms. Participants can push one of four buttons on the keyboard which corresponds to one of the four match figures in the lower half of the screen. During one single task run, participants have to match either according to color or shape. The color-shape combinations are shown in a way that there is one ‘right’ option. Half of all task runs consisted of ‘switch’ runs, in which the type of cue differs from the previous run. The other half consists of ‘repetition’ runs, in which the type of cue is identical to the previous run. The duration of the test is approximately six minutes. Both the mean reaction time for switch runs and repetition runs will be used as an outcome measure for switching (for more detailed information about this task, see [70]).

*Inhibition* addresses one’s capability to deliberately inhibit dominant and automatic responses to certain stressors [68]. Inhibition will be measured with the Sustained-Attention-to-Response Test (SART [72]). During this test, digits (ranging from 1–9) are displayed one-by-one in the centre of the screen. Participants are instructed to push a button on a button-box when a digit appears on the screen, except when the digit is a ‘3’, which occurs in 11.1 % of the cases. A total of 450 digits will be presented, each with a duration of 250 ms. The interval between digits is 850 ms. The number of inhibition errors (thus, when a participant presses the button when a ‘3’ appears on the screen), will be taken as a measure for inhibition.

To obtain more insight into cognitive functioning, we will additionally evaluate the *subjective costs* (fatigue, motivation, demands, and effort) associated with doing these tests [73, 74]. These *subjective costs* will be measured using single item measures, answered on a scale from 1 (*not at all*) to 10 (*very much*). Before doing the cognitive tests, participants will rate how motivated they are to do the tests. Fatigue will be measured prior to and after the tests. In addition, after each cognitive test, participants will be asked to score how demanding the test had been. After completing all the tests, participants indicate how much effort they spent when doing the tests.

#### Aerobic fitness

We will measure aerobic fitness using the estimated maximal oxygen uptake (VO_2max_), obtained from the UKK walk test [75]. This test has found to be valid and feasible for a healthy adult population [75]. During this test, participants have to walk 2 km as fast as possible. The test will take place on a 400 m outdoor track in groups of (maximum) ten participants. Participants start individually every 30s. The instruction is as follows: “Walk the distance as fast as you can, but do not risk your health”. Immediately after the walk, the heart rate and walking time will be measured. The variables used to calculate VO_2max_ are walking time, heart rate, body weight, height, and gender (see [75] for exact equations).

We will also measure participants’ *subjective costs* of doing the UKK walk test. The questions used for this purpose are similar to those asked before and after the cognitive tasks, except that we add another question about how ‘short of breath’ participants are immediately after the test. Completion of the test, including filling out the items about subjective costs, takes about 25 min.

### Weekly measures during the intervention period

During the six weeks of the intervention period, each Wednesday all participants (intervention and control condition) will be requested to complete a short questionnaire that will be sent by email. Completion of the weekly measures takes five minutes.

#### Employee well-being

We will use twelve single-item measures as indicators of employees’ *weekly well-being* in order to obtain a detailed account of the course of these indicators during the intervention period. Single item measures are chosen because such measures require minimum effort to complete, and because they have been found to be valid indicators of employee well-being [76]. The items are introduced as follows: “Can you indicate with a report mark between 1 (not at all applicable) to 10 (extremely applicable) to what extent the following state of minds were applicable to you during the previous two days?” The items are: “healthy”, “tired”, “tense”, “happy”, “satisfied”, “energetic”, “stressed”, “vital”, and “irritated”. The response scale from 1 to 10 is based on the typical Dutch grade notation system.

Additionally, three single item measures to assess participants’ *weekly self-efficacy* regarding exercise, work, and other private personal goals will be as follows: “Can you indicate with a report mark between 1 (not at all certain) to 10 (extremely certain) how certain you are that you can reach your goals regarding “exercise”, “work”, “other private personal goals” during the previous two days?

Furthermore, to measure employees’ *weekly sleep quality*, the 6-item sleep quality scale derived from the Dutch Questionnaire on the Experience and Evaluation of work will be used [63]. As these items were originally developed for chronic sleep complaints, the scale is adapted for weekly measurement. An example item is: ‘I slept well last week’ (reversed; 1 = *yes*, 0 = *no*). Next to sleep quality, also the mean hours sleep a night will be assessed (i.e. ‘*sleep quantity’*).

#### Exercise activities

Each week, participants’ exercise and physical activity levels will be assessed. Participants in the exercise condition are requested to indicate their *compliance* to the guided and individual running sessions. Additionally, if applicable, they are asked to indicate whether they performed a missed guided running session on their own, and whether they performed this session according to the fixed running schedule or not. Besides that, they are requested to indicate if they performed *other exercise activities* than running during the past week (type, duration, and frequency). Participants in the control condition will be asked to indicate whether they engaged in exercise activities during the past week (type, duration, and frequency). Finally, all participants are requested to report their *physical activity* during the past week: ‘On how many days a week were you physically active during at least 30 min a day (only count physical activity that is equally demanding as brisk walking or biking. Activities shorter than ten minutes do not count) – during your work and free time together?’ (*0-7 days*) [77].

#### Subjective exercise experiences

To measure how participants in the exercise intervention experience the running sessions, we will administer single-item measures about *pleasure* and *effort.* The items are introduced as follows: “Can you indicate with a report mark between 1 (not at all applicable) to 10 (extremely applicable) how you experienced last week’s running sessions?” Separate ratings will be collected for the guided and individual running sessions. Additionally, the extent to which employees can ‘unwind’ from work during the running sessions will be measured using an adapted version of the 4-item *psychological detachment* scale of the Recovery Experience Questionnaire [78]. An example item is: ‘Last week, I forgot about work during the running sessions’. Previous research shows that psychometric properties are good [78].

### Statistical analyses

#### Sample size

Based on previous research, it is difficult to predict an exact effect size on which we can base our sample size, given that these studies report small to large effect sizes [19, 20, 22, 23], and that they differ in design (i.e., duration of exercise intervention; absence of control condition) and target audience (i.e., low to clinical levels of work-related fatigue). Since we conduct our study among employees with high but no clinical levels of work-related fatigue (we expect a decrease in work-related fatigue, but not as large as we would expect among employees with clinical levels) and our intervention is relatively short, we expect a small to medium effect size. To determine sample size, a power analysis has been conducted in the statistical program G*power [79]. This analysis was based on a repeated measures ANOVA with work-related fatigue as within subjects factors and condition as between subjects factor. This analysis showed that a total of 90 participants was required in order to detect a small to medium effect of .15 on the primary outcome work-related fatigue from pre to post intervention, given a two-sided 5 % significance level, a power of 80 %, and an correlation of .05 across repeated measures [80]. Because we anticipated a dropout rate of about 20 %, we intend to recruit 108 participants.

### Analyses of primary and secondary outcomes

We will use intention-to-treat analysis, meaning that all participants who are randomized to a condition will be included in the analyses. To test the efficacy of the exercise intervention (i.e. change in primary and secondary outcomes from pre [T0] to post [T6]), we will perform 2x2 repeated measures (M)ANOVAs with condition (exercise versus wait list) as between-subjects factor, and time (pre [T0] versus post [T6]) as within-subjects factor. We will conduct a RM-MANOVA for the three indicators of work-related fatigue together (*Hypothesis 1*). Furthermore, we will conduct separate RM-(M)ANOVAs for respectively self-efficacy (*Hypothesis 2a and 2b*), sleep (*Hypothesis 2c and 2d*), work ability (*Hypothesis 2e*), cognitive functioning (*Hypothesis 2f*), and aerobic fitness (*Hypothesis 2 g*). The Matching Task will be analyzed using a 2x2x2 mixed design ANOVA, with ‘run type’ (switch versus repetition), and time (pre versus post) as within-subjects factors, and condition as between-subjects factor (exercise versus wait list). In all analyses, we are particularly interested in the ‘condition x time’ interaction, because this indicates that the conditions differ from each other with respect to the development of the outcome measure over time. If applicable, significant interactions will be further examined by paired-sample t-tests. Partial eta-squared will be reported as effect size.

Additionally, to obtain more thorough insight into the clinical meaningfulness of the changes brought about by the exercise intervention [81], we will perform a Chi-Square Test to see if the number of participants who score below cut-off scores of fatigue after the intervention period (T6) differs between the intervention and the control condition (emotional exhaustion <2.2 on the UBOS; overall fatigue: <22 on the FAS).

To investigate whether intervention-effects last during the follow-up period, for each primary and secondary outcome measure, a repeated measures MANOVA will be performed to see whether it differs between pre (T0), post (T6), 6 weeks after the intervention period (T7), and 12 weeks after the intervention period (T8). If the overall time effect of MANOVA is significant, post hoc tests will be carried out to exactly determine between what time points the outcome had changed. As follow-up measures are only available for the intervention condition, the control condition will not be included in these analyses.

### Analyses of weekly trajectories of employee well-being

The trajectories of employees’ weekly well-being during the intervention period will be analyzed using growth models in a multilevel modeling framework, because our weekly occasions (level 1) are nested within persons (level 2). This analyzing method allows us to estimate inter-individual as well as intra-individual patters of change over time in a powerful way [82]. The data will be analyzed using MPlus [83]. For each of the weekly indicators of employee well-being, a model will be tested. This model consists of an intercept term, and variances at the occasion and person level. Furthermore, ‘time’ (7 occasions: T0 to T6) will be added as a random factor to the model (to measure the rate of change per unit of time), as well as ‘time^2^’ (to measure a change in the rate of growth). Additionally, ‘condition’ (0 = control, 1 = exercise) will be added as a fixed factor to the model, and will interact with ‘time’, and ‘time^2^’. As such, it will be investigated whether there is an effect of exercise on the rate of change in well-being over time.

### Analyses of moderators

To assess to what extent the exercise experiences (‘pleasure’, ‘detachment’, ‘effort’) and compliance (number of attended running sessions) of participants in the exercise condition moderate the weekly outcomes of employee well-being, we will also use a multilevel modeling framework performed in MPlus [83]. For each indicator of employee well-being in relation to the exercise experiences and attendance rate, we test a model. This model is equivalent to our first model, except that condition is not a predictor anymore (because only participants in the exercise intervention are studied), and the three exercise experiences and compliance are added as fixed covariates in the model and interact with ‘time’, and ‘time^2^’.

## Discussion

This article describes the design of a two-arm RCT to evaluate the efficacy of an exercise intervention on work-related fatigue. Except for work-related fatigue, the effect of exercise on five secondary outcomes related to work-related fatigue will be studied. Furthermore, we will gain insight in how different indicators of employees’ well-being develop during the intervention period. This will inform us if, and if so, at what point in time the intervention is effective in changing these indicators. Furthermore, we also examine participants’ experiences of and the attendance to the running sessions of the exercise intervention to get a better understanding of the extent to which these experiences and activities may have differential effects on intervention efficacy.

Since we wish to investigate the effect of exercise compared to the natural course of work-related fatigue over time, and there is no standard effective intervention for work-related fatigue available yet, we choose for a wait list as control condition. To overcome possible ethical and practical concerns of wait lists in randomized controlled trials [84], we decide to employ an exercise intervention of a relatively short duration (i.e. six weeks). It could be imagined that withholding an intervention from employees with high levels of work-related fatigue for a longer period of time could worsen their problems. Furthermore, a longer waiting period for the participants in the control condition could result in higher attrition rates.

### Strengths and limitations

The main strength of this study is its methodological quality: the use of the randomized controlled design, intention-to-treat analysis, follow-up measures, and multi-method measures (self-reports and performance measures). Using these design elements reduces several sources of bias, like selection bias, withdrawal bias, and allows us to make strong causal inferences [85]. As such, the current RCT will add to the existing scientific literature about the effect of exercise on work-related fatigue. Since our intervention will be delivered in a uniform fashion to a specific homogeneous target audience, and thus can be regarded as an ‘efficacy trial’ (i.e., the intervention produces the expected result under ideal circumstances), the results of the current study may also provide a basis for future ‘effectiveness’ trials in which the (implementation of the) intervention can be investigated among a broader defined population in a less standardized way [86].

Another strength is our sample. We choose to investigate employees who are still able to work, but have (relatively) high levels of work-related fatigue. As such, our intervention can be regarded as secondary prevention [87]. We expect that exercise not only reduces work-related fatigue symptoms, but also prevents these from becoming more severe (i.e. clinical burnout [60]). We believe that it is valuable to target these employees, because prevention is better than cure. However, future research could also investigate whether exercise benefits clinical burnout.

Despite its strengths, several issues concerning our study deserve attention. First, our study is not blinded, because active participation of the participants and trainers is necessary in our intervention. Furthermore, because the number and timing of outcome measures differed between the intervention and the control group, it was not possible for researchers involved in this study to be blind to the condition they were assessing. Although blinding is preferable, it should be noted that our primary and secondary outcomes require no subjective evaluation from the outcome assessors. As such, we argue that this issue is of limited influence [88].

Additionally, the fact that the control group in the current study is on a wait list needs attention. Although a wait list condition is – given our research aims and current evidence about effective interventions for work-related fatigue – the best option, it has been argued that wait list groups are not truly untreated because they are contacted, consented, randomized, and measured [89]. This might cause a decrease in symptoms while not receiving an ‘active’ intervention. Following this reasoning, the effects that may be found in this study may be underestimations of the true causal effects.

Lastly, we will not use objective measures to watch participants’ exercise intensity (e.g. heart rate monitoring), although these measures provide the most accurate estimate of participants’ exercise intensity. However, it has been shown that being able to talk is well correlated with heart rates that match low intensity [52], suggesting that it is an effective tool to monitor exercise intensity. Furthermore, the trainers are instructed to watch participants’ intensity, by making sure they can still talk while running. To control whether the running was not too intensive, we will ask participants about their perceived exertion during the running sessions. Although this is a subjective measure, it provides a fairly good estimate of the actual spent effort during physical activity [90].

### Implications for practice

This RCT is relevant, because work-related fatigue is prevalent among employees and it negatively impacts employees’ health and work performance. If the intervention is proven effective, this would suggest that there is a relatively simple, inexpensive and accessible intervention strategy to reduce work-related fatigue. The results of this RCT could be used to provide better evidence-based policies and practices to employees, employers, health practitioners, and policy makers concerning the effect of exercise on employee well-being.

## Conclusion

This RCT has the ability to make a contribution to the evidence base for the effect of exercise on work-related fatigue.

## Abbreviations

RCT: Randomized controlled trial
